# Polymer-Bitumen Interaction: A Correlation Study with Six Different Bitumens to Investigate the Influence of SARA Fractions on the Phase Stability, Swelling, and Thermo-Rheological Properties of SBS-PmB

**DOI:** 10.3390/ma14051273

**Published:** 2021-03-08

**Authors:** Martin Wieser, Andreas Schaur, Seraphin Hubert Unterberger

**Affiliations:** Unit of Material Technology, University of Innsbruck, Technikerstrasse 13, 6020 Innsbruck, Austria; m.wieser@uibk.ac.at (M.W.); andreas.schaur@student.uibk.ac.at (A.S.)

**Keywords:** polymer modified bitumen, SBS, bitumen composition, polymer compatibility, rheological properties

## Abstract

The aim of this work is to determine the influence of the bitumen chemistry on the rheological performance of bitumen and polymer modified bitumen (PmB), as well as the polymer distribution and storage stability. Six different bitumens and their 5 wt.% SBS mixtures are considered in this work. The bitumen composition was determined by SARA fractioning, which was then correlated with the glass transition temperature, complex modulus |G*|, and phase angle, which were obtained by parallel-plate dynamic shear rheology in the temperature range of −25 to 65 °C. The polymer distribution, which was derived from fluorescence microscopy images and the storage stability (determined by tube test) also correlated with the SARA fractions. It was found that the saturates decrease |G*| and T${}_{g}$ and increase the phase angle in crude bitumen, while the asphaltenes increase |G*| and the phase angle. For PmB, the amount of swelling was determined by the saturate content of bitumen. The glass transition temperature of PmBs increases for low saturate and decreases for high saturate contents. |G*| and the phase angle of PmBs correlates with the saturate content, with a varying influence depending on a high or low saturate content and the temperature range due to saturate depletion in the bitumen-rich phase and the varying vol% polymer-rich phase. The aromatic and resin fractions show no correlation in the considered bitumens and PmBs.

## 1. Introduction

Bitumen is readily available as a by-product of the crude oil refining process. Since it exhibits convenient mechanical properties, it is widely used as a binder material for paving and sealing applications. Due to the multitude of different molecules present in bitumen, an exact chemical characterization is difficult. Therefore, it is common to refer to the SARA fractions when talking about the chemistry of the bitumen composition. These fractions represent classes of compounds with similar physicochemical properties such as solubility, aromaticity, molecular weight, and polarity [1,2,3]. In principle, the ratio of the SARA fractions should therefore be able to be used to predict the thermo-mechanical properties of bitumen [4]. Radenberg et al. successfully correlated the asphaltene content with the softening point and phase angle [5]. Weigel et al. found an increasing penetration index, increasing complex modulus |G*|, and decreasing phase angle with asphaltene content [6]. In a subsequent work, Weigel et al. used principal component regression to successfully correlate the bitumen composition and the average molecular weights with the softening point, penetration index, complex modulus |G*|, and phase angle [7]. To improve the mechanical and aging related properties, bitumen is often modified with polymers [2,3] or inorganic compounds [8,9,10]. Especially the influence of aging or extreme temperatures on the microstructure and rheological properties are ongoing fields of research [11,12,13,14,15,16,17,18,19,20]. Studies were performed to investigate the interaction of bitumen and SBS, focusing on the SBS structure [21] and resulting thermal properties of SBS/bitumen blends [22]. However, no studies have demonstrated the correlation of the bitumen composition with the rheological properties of PmB. The aim of this work is to extend the research of Weigel et al. [7] to polymer modified bitumen and to gain further insights into the connection of the bitumen chemistry, the bitumen rheology, and the polymer-rich phase stability. The considered polymer-bitumen mixtures were prepared as 5 wt.% mixtures with a branched SBS (Kraton D 1184), which is commonly used for polymer modified bitumen. Six different bitumens with varying origins and penetration grades were used in this work.

## 2. Materials and Methods

### 2.1. Methods

**Figure 1:**
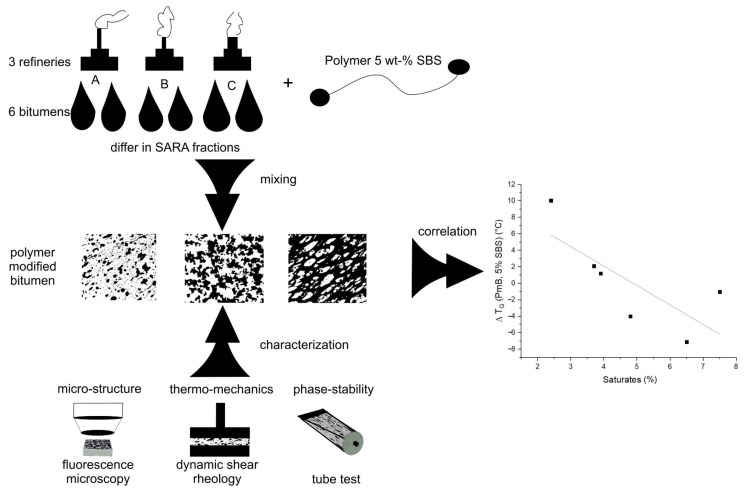
Schematic diagram indicating the material preparation and characterization.

In this study, the properties of six different PmBs were analyzed using dynamic shear rheometry (DSR), fluorescence microscopy, and the tube test. The results of the characterization were then used to correlate the phase stability, polymer swelling, and thermo-rheological properties with the SARA fractions of the respective bitumen. Schematic information on the method is reported in Figure 1.

*Mixture preparation*: For the optimal dispersion of the polymer in bitumen, the following routine was applied. After preheating bitumen in an oven (160 °C), the mixture was transferred to a heating plate and continuously stirred with an IKA T50 digital Ultra Turrax running at 2000–3000 rpm. The polymer was added at 180 °C. In order to avoid the decomposition of the bitumen components, the temperature did not exceed 200 °C. After adding the target amount of polymer, the mixture was dispersed at 190 °C for 60 to 90 min. Mixing was deemed complete when the polymer phase was homogeneously distributed in bitumen or no change in structure was observed in fluorescence microscopy for 15 min. The temperatures, mixing time, and mixing speed were chosen to account for workability (the viscosity of the mixture has to be low enough to be workable), degradation temperature (bitumen should not be heated over 200 °C [23]), and polymer degradation due to shear stress [24].

*Fluorescence microscopy*: The polymer distribution in PmB was measured with an Axio Imager A.2m fluorescence microscope from Zeiss optics equipped with a 100 watt high-pressure mercury plasma arc-discharge lamp (HBO 100). The objective used was a Zeiss EC Epiplan 40×. For better contrast, a green color filter was applied. The samples shown in the Results Section were taken and analyzed directly after dispersing the polymer in bitumen. Sample preparation was carried out according to the freeze-fracturing method described in ÖNORM EN13632.

*Tube test*: The storage stability of PmBs was determined with a tube test according to ASTM D7173. Values are reported as ring-ball softening point temperatures for the upper third and lower third of the tube after conditioning.

*DSR*: For the thermo-mechanical characterization of the polymers, bitumens and PmBs, a HAAKE MARS III parallel plate rheometer in forced oscillation mode was used. A plate geometry of 8 mm in diameter was used. The amplitude of the maximum strain and the angular frequency were set in accordance to Superpave AASHTO T315 ASTM D7175 (0.001 maximum strain and 10 Hz angular frequency). Samples were obtained by casting the sample geometry in silicone molds. The glass transition temperature was determined as the maximum of G″.

### 2.2. Materials

*Bitumen*: The investigated bitumens originated from three different refineries (A, B, C) and were chosen such that they varied in their needle-penetration grade and composition. The considered bitumens, penetration grades, and SARA fractioning composition (by thin-layer chromatography coupled with a flame ionization detector) can be found in Table 1.

**Figure 2:**
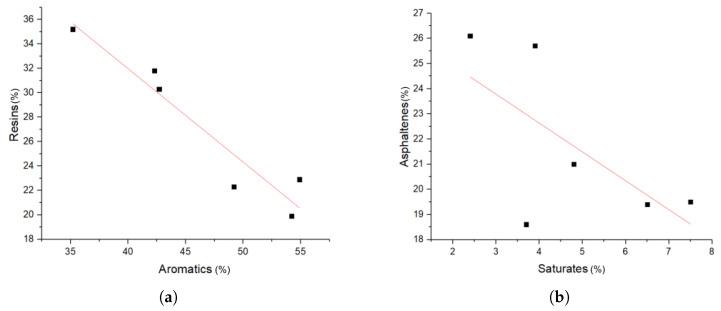
Correlation of SARA fractions for crude bitumen. (**a**) Resin vs. aromatics and (**b**) asphaltene vs. saturate content.

Although this selection was broad, there still were some correlations between the fractions, which had to be considered in the interpretation of the results. The aromatics and resin fractions were highly correlated, and the saturates and the asphaltenes were slightly correlated, as can be seen in Figure 2. The latter correlation might be explained by the production process, since harder bitumens are generally exposed to higher temperatures and lower pressures, yielding bitumens with higher asphaltene and lower saturate content [1]. These correlations have to be taken into account when discussing the results. The other fractions did not show any correlation.

## 3. Results and Discussion

### 3.1. Crude Bitumen

**Figure 3:**
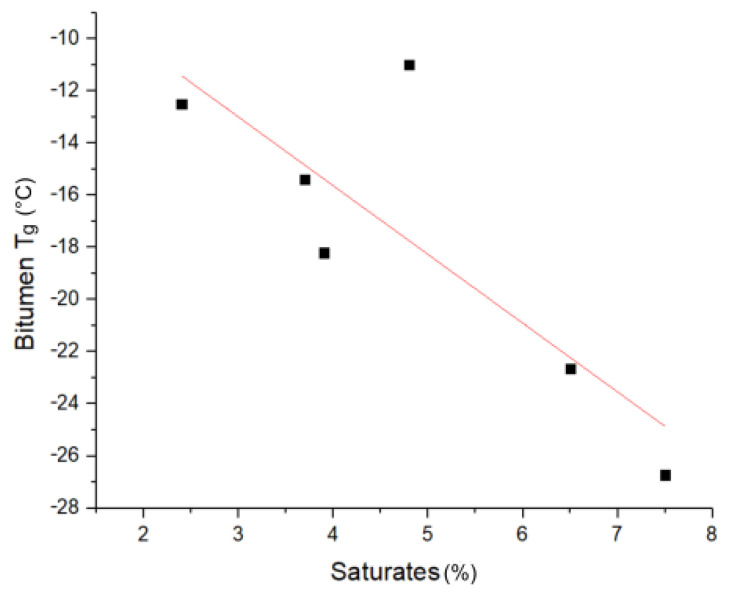
Bitumen glass transition temperature (T${}_{g}$) correlation with saturate content.

*Glass transition temperature (T${}_{g}$)*: The influence of the saturate content on the glass transition temperature of bitumen can be seen in Figure 3. The observed glass transition temperature ranges from −11.0 to −26.7 °C. The glass transition temperature shows a reasonable correlation with the saturate content (more saturates equals lower T${}_{g}$) and no correlation for the remaining fractions of the considered bitumens. Jimenéz-Manteos et al. reported a correlation of the glass transition temperature with the crystalline fraction of maltenes [25]. Masson et al. found that the main glass transition temperatures of the isolated fractions were −60 °C (saturates), −15 °C (aromatics), 25 °C (resins), and 70 °C (asphaltenes) [26]. Not considering interactions, saturates and aromatics should therefore lower the T${}_{g}$, while resins and asphaltenes should increase the T${}_{g}$. The inverse correlation of the aromatics and resin fraction in the considered bitumen samples therefore may cancel out the effect on T${}_{g}$, which however may still be present. The lack of influence of the asphaltenes fraction on T${}_{g}$ may be explained by the commonly used colloidal model of bitumen, where the asphaltenes are dispersed as micelles in the maltene matrix, thus not contributing to the T${}_{g}$ of the matrix [2].

**Figure 4:**
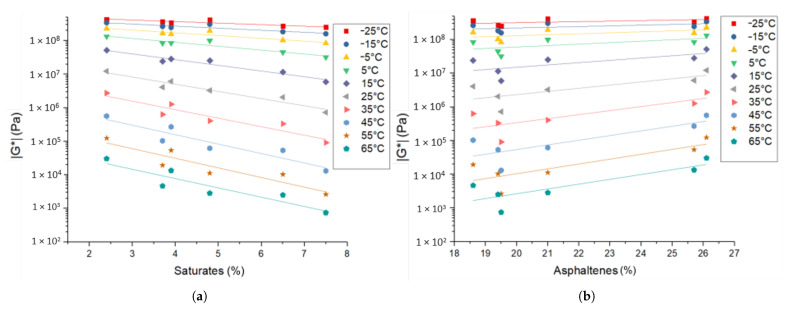
Bitumen |G*| correlation with (**a**) saturate and (**b**) asphaltene SARA fractions.

*Rheometry*: The investigated rheological parameters are the complex modulus |G*| and the phase angle δ. The influence of the bitumen composition on |G*| is shown in Figure 4. It can be seen that an increased saturate content yields a decrease of |G*|. Lu et al. and Laukkanen et al. established a relationship between |G*| and the average molecular weight of oils and binders, which explains the effect of the saturates (lower molecular weight) [18,27]. Furthermore, an increase of the asphaltene fraction results in an increase of |G*|. Similar results have been found by Weigel et al. for the asphaltene content [6]. Both the effects of the saturates and the asphaltenes are more pronounced for higher temperatures. No influence of the aromatics and resin fraction on |G*| was found in the considered bitumen samples, which may be due to them having no significant effect or the influences canceling each other out since those fractions are correlated. The latter effect is more plausible, since the opposing influences of resin and aromatics content on |G*| were found by Sultana et al. [28].

**Figure 5:**
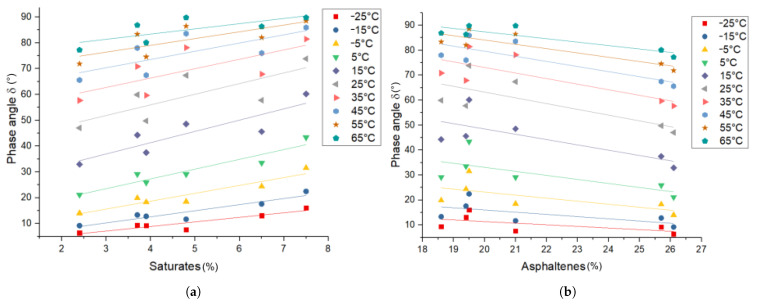
Bitumen phase angle δ correlation with the (**a**) saturate and (**b**) asphaltene SARA fractions.

The influence of the bitumen composition on the phase angle δ can be seen in Figure 5. An increase of the saturate content results in an increase of the phase angle, while an increase of the asphaltene content results in a decrease of the phase angle. The aromatics and resin content is not correlated with the phase angle. This can be explained similarly to |G*|, with a lower molecular weight leading to a less viscous behavior [6,27] and the effects of aromatics and resins most likely canceling each other out [29].

### 3.2. Polymer Modified Bitumen

**Figure 6:**
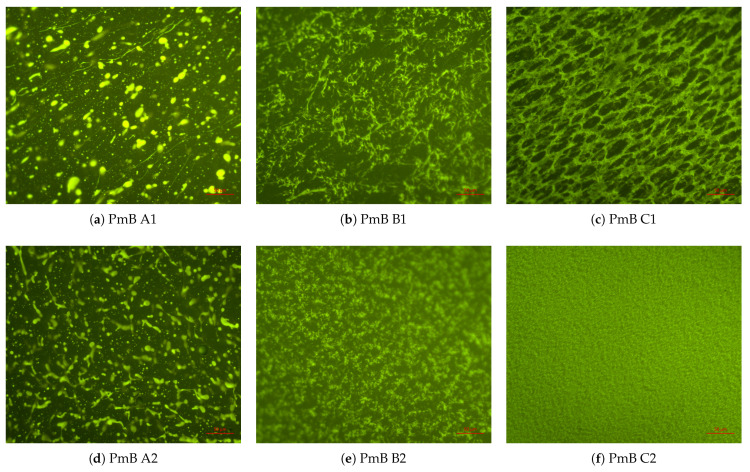
Fluorescence micrographs of different bitumens modified with 5 wt.% SBS at 40× magnification. PmB, polymer modified bitumen.

**Figure 7:**
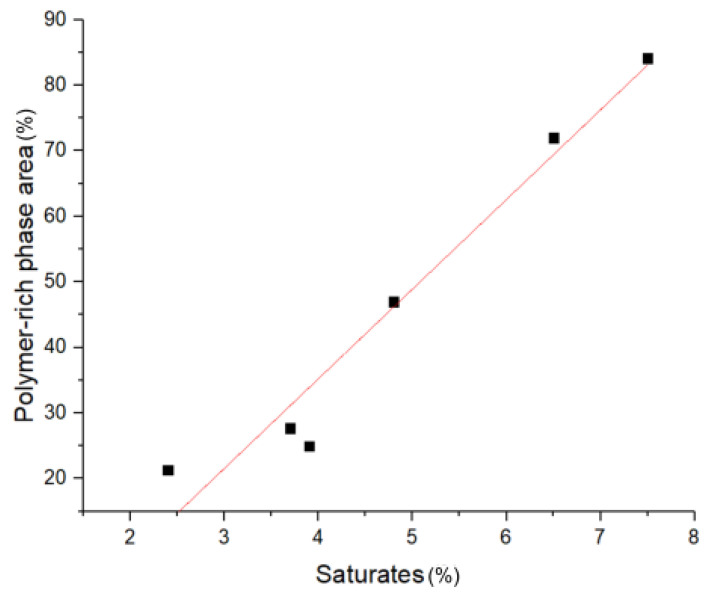
Polymer-rich phase area vs. saturate content. All PmBs are modified by 5 wt.% SBS.

**Figure A1:**
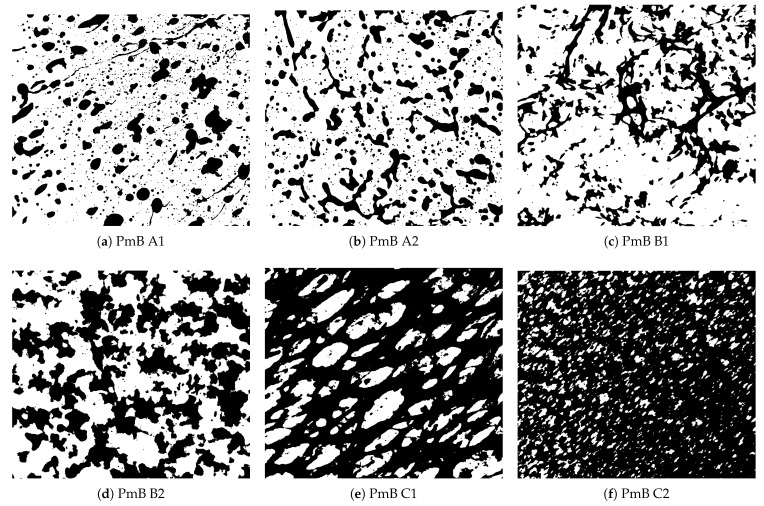
Thresholded fluorescence micrograms of different bitumens modified with 5 wt.% SBS. Magnifications vary, since only image sections could be considered due to illumination differences. A representative part of the image was chosen. The original image magnification is 40×.

*Polymer distribution*: The compatibility of a polymer with a certain bitumen can be established by two factors: (a) the amount of swelling (e.g., volume increase of the polymer-rich phase due to the incorporation of bitumen components) and (b) the thermal stability of this mixture. Fluorescence microscopy images to evaluate (a) are depicted for all 5 wt.% mixtures in Figure 6. To evaluate the degree of swelling, the pictures were thresholded, and the area of the polymer-rich phase was measured, the polymer-rich phase being green (for the thresholded images, see Appendix A, Figure A1). As depicted in Figure 7, it can be seen that swelling occurs in all considered bitumen mixtures, and the degree of swelling increases with increasing saturate content, with phase inversion occurring slightly over 5 wt.% saturates. This is most likely due to the saturates being incorporated in the polybutadiene section of the polymer due to chemical similarity. No correlation was found with the aromatics fraction, contrary to the literature, where the aromatics fraction is reported to be important for compatibility [2]. The limited influence of the aromatics fraction can be explained by limited swelling of the polystyrene domains, which was also observed by Fawcett et al. [30]. The stabilizing effects of aromatics and resins on the PmB microstructure are however still possible.

Furthermore, it can be seen that a low saturate content results in the polymer-rich phase being distributed in a bitumen-rich matrix (Figure 6: PmBs A1, A2, and B1), while a high saturate content results in the bitumen-rich phase being distributed in a polymer-rich matrix (Figure 6: PmBs C1 and C2), which is also called phase inversion. PmB B2 shows equal amounts of the polymer-rich phase and bitumen-rich phase, yielding no distinctive matrix phase.

**Figure 8:**
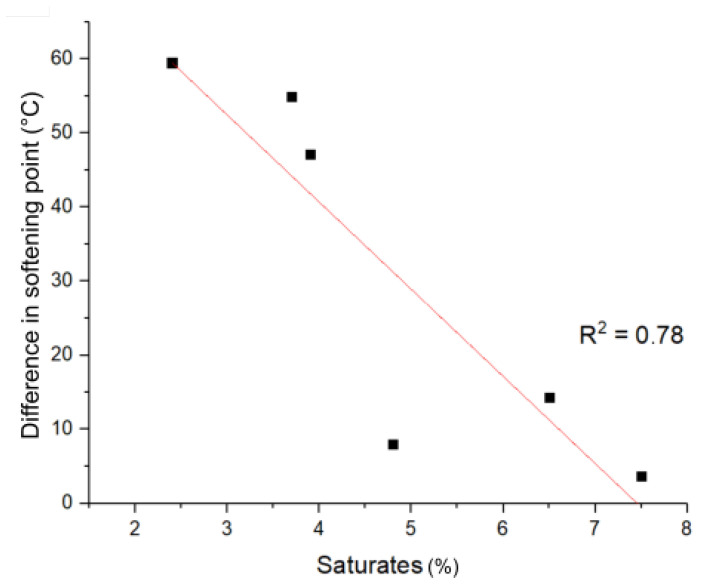
Difference in the softening point (top-bottom) for the tube test of the considered PmBs vs. saturate content.

*Phase stability—tube test*: The influence of the saturate content on the phase stability of the respective PmBs is shown in Figure 8. It can be seen that the difference in the softening point (top-bottom) is higher for lower saturate content. This can be explained by an increased amount of the less dense polymer-rich phase in the upper region of the tube, thus suggesting the low temperature stability of these mixtures. PmBs with higher saturate content exhibit lower differences in softening points, which suggests comparatively better thermal stability [3]. No significant correlations were found for other fractions. These results are consistent with the polymer distributions observed in Figure 6. This suggests that the phase distributions at elevated temperatures are either stable or more favorable for the polymer-rich phase. Therefore, PmBs with phase inversion (C1, C2) or equal phase proportions (B2) exhibit a stable phase distribution, and non-phase inverted PmBs (A1, A2, B1) show phase segregation in the tube test due to buoyancy effects.

**Figure 9:**
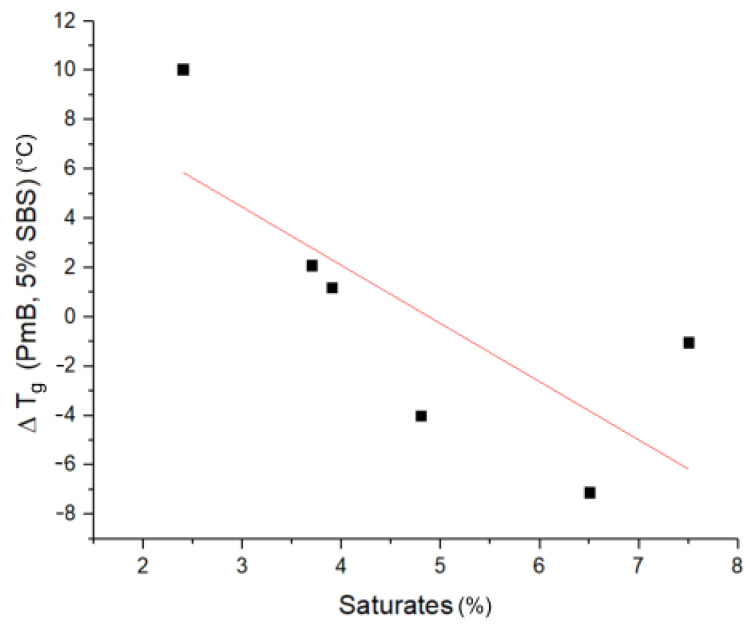
ΔT${}_{g}$ of PmB compared to the respective crude bitumen vs. saturate content.

*Glass transition temperature (T${}_{g}$)*: In Table 2, the glass transition temperatures of the crude bitumen and the polymer modified bitumen can be seen, while in Figure 9, the difference in the low temperature glass transition of the crude bitumen and the respective PmBs (ΔT${}_{g}$) is depicted. Low saturate content leads to an increased T${}_{g}$ of PmB compared to crude bitumen. This can be explained by the saturates being incorporated into the the polymer-rich phase, leading to a bitumen-rich phase being depleted of low molecular weight components, which in turn increases the T${}_{g}$ of this phase [25]. Since the bitumen-rich phase is the matrix phase in these PmBs, this increases their T${}_{g}$. At higher saturate content, a reduction of T${}_{g}$ is observed. This can be attributed to the polymer being swollen mainly by the saturate fraction. The polybutadiene section of the SBS polymer exhibits a lower T${}_{g}$ than the crude bitumen [22]. Therefore, since the polymer-rich phase is the matrix phase in these PmBs, their T${}_{g}$ is lower.

No correlation of ΔT${}_{g}$ and the aromatics, resin, and asphaltene fraction is visible in PmB, which suggests that the interaction of these fractions with the polybutadiene phase is negligible or canceled out due to their correlation with each other [28].

**Figure 10:**
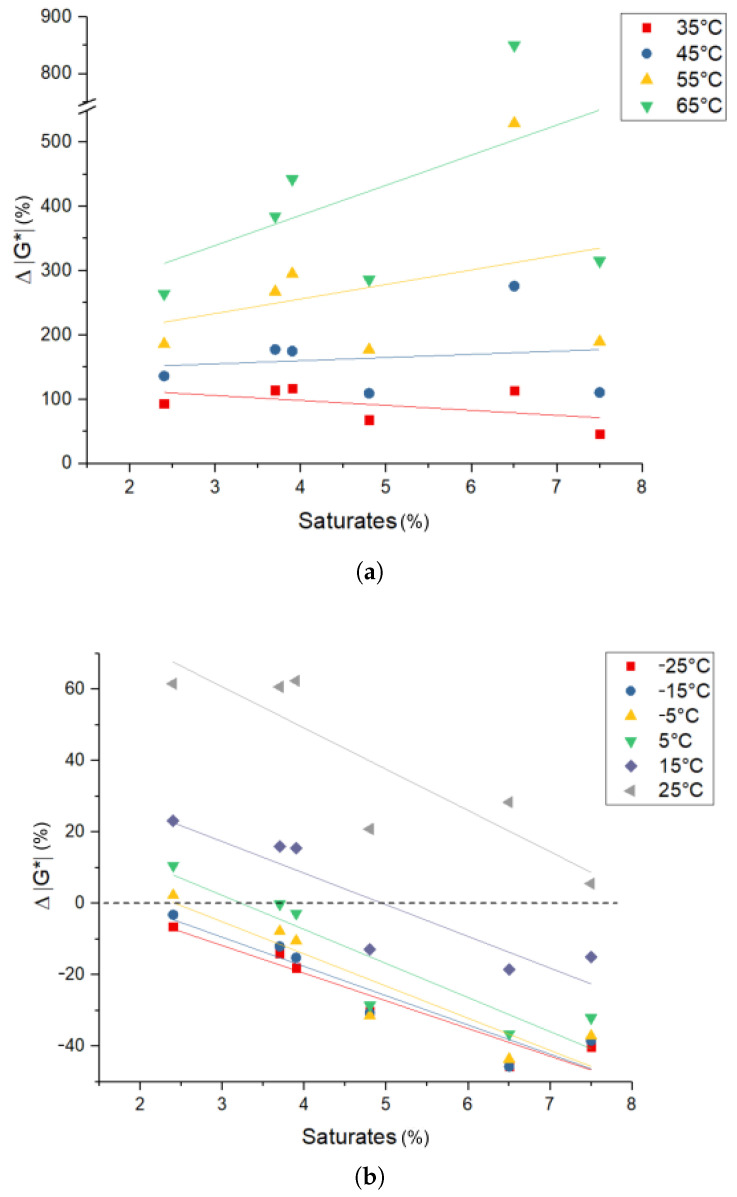
Δ|G*| of PmB compared to the respective crude bitumen vs. saturate content.

Rheology—complex modulus |G*|: In Figure 10, the influence of the bitumen composition on |G*| of PmB in comparison to the respective base bitumen can be seen for temperatures ranging from −25–65 °C (Δ|G*| (%) = (|G*${|}_{PmB}-|$G*${|}_{Bitumen}$)/|G*${|}_{Bitumen}\;\cdot \;\;100$). At temperatures above 65 °C, |G*| of the base bitumens cannot be accurately determined with the geometry used due to insufficient torque. At the lowest considered saturate content, |G*| increases for temperatures above −15 °C, while for −25 and −15 °C, a slight decrease can be seen. At the highest considered saturate content, |G*| decreases for temperatures below 15 °C and increases for higher temperatures. This increase with temperature is more pronounced for higher saturate contents than for lower saturate contents. The bitumens with intermediate saturate content roughly follow a linear trend. Therefore, Δ|G*| decreases with higher saturate content in the temperature range from −25 to −35 °C and increases for higher temperatures.

Based on the obtained results, three effects are identified, which vary in significance depending on the microstructure of PmBs. In the case the bitumen-rich phase is the matrix phase (low saturate content), changes to the bitumen-rich phase are the most relevant. In general, the bitumen-rich phase |G*| increases due to the depletion of saturates, which migrate into the polymer-rich phase [6,18,27]. This effect can be seen most prominently in A1 (2.4% saturates), where the |G*| of the mixture is higher than the crude bitumen |G*| for temperatures higher than 5 °C, even though the |G*| of the polymer is lower than the crude bitumen up to 20 °C (see Table 3). A similar effect can be seen for A2 and B1, although less pronounced due to the increased amount of the polymer-rich phase. In case the polymer-rich phase is the matrix phase (high saturate content), the properties of the polymer rich phase are the most relevant. Since |G*| of the polymer is lower than the |G*| of bitumen at lower temperatures, the |G*| of the polymer-rich phase (and therefore, the |G*| of PmB since it is the matrix phase) is also lower than the |G*| of bitumen. Additionally, saturate incorporation into the polymer-rich phase decreases its |G*| at lower temperatures, since the saturates exhibit a lower |G*| compared to SBS. This is evident in PmB C2, where the PmB |G*| is 32% lower at 6 °C compared to the point of equal |G*| of the single components at 6 °C (see Table 3).

At higher temperatures, |G*| of the polymer is higher than the crude bitumen (see Table 3). This difference increases with temperature (|G*| of the polymer diminishes less with temperature). Therefore, the PmB Δ|G*| increases with temperature. This effect is present in all PmB mixtures; however, it is more pronounced for PmBs with a greater polymer-rich phase (higher saturate content), which causes the change in slope, as can be seen in Figure 10.

Aromatics, resin, and asphaltene contents show no correlation at low temperatures and no significant correlation at higher temperatures, which may be due to the effect of their canceling out.

**Figure 11:**
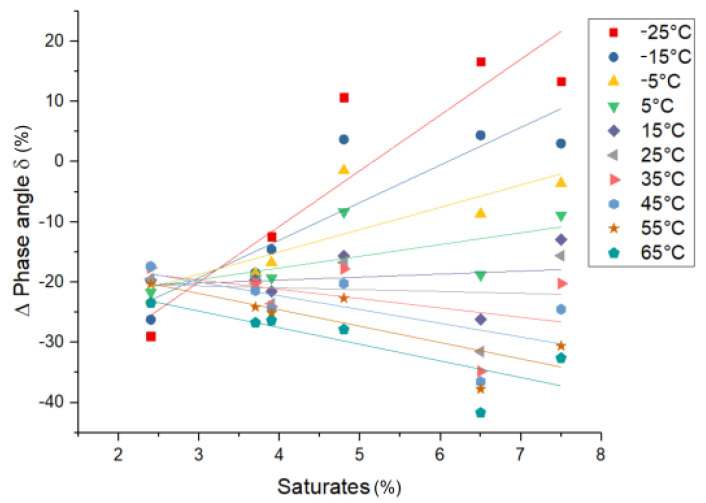
Δ phase angle of PmB compared to the respective unmodified bitumen vs. saturate content.

*Rheology—phase angle δ*: The influence of the saturate content on the phase angle of PmB in comparison to the base bitumen (Δδ (%) = ($\delta_{PmB}-\delta_{Bitumen}$)/$\delta_{Bitumen}\;\cdot \;\;100$) can be seen in Figure 11. The pure polymer exhibits a phase angle smaller than bitumen at all temperatures. At low saturate content, the polymer-rich phase acts like an elastic filler at all temperatures, thus reducing the phase angle. Additionally, the decrease of the saturate content in the bitumen-rich phase due to migration into the polymer-rich phase causes a relative increase of the asphaltene fraction in the bitumen-rich phase, which is also linked to a lower phase angle (see Figure 5). At higher saturate content, the effect on δ increases, which can be linked to the increased vol% of the polymer-rich phase (swelling). At lower temperatures (−25–5 °C), the polymer-rich phase increases the phase angle. This can be explained by the incorporation of saturates, which shift the combined T${}_{g}$ of these two components to lower temperatures (T${}_{g,Sat}$ = −60 °C [26]) and therefore increase the phase angle. At higher temperatures (25–65 °C), the polymer-rich phase decreases the phase angle, since the bitumen-rich phase is increasingly liquid, and the polymer-rich phase retains elasticity until the polystyrene domains melt at their glass transition temperature (T${}_{g},Polystyrene$ = 100 °C). The correlation of the asphaltene, aromatics, and resin fractions with the phase angle is found to be not significant in the considered data set.

## 4. Conclusions

The bitumen composition determined by SARA-fractioning was successfully correlated with the T${}_{g}$, |G*|, phase stability, and phase angle for both unmodified and SBS modified bitumen. The following effects were found:

Bitumen:

- A higher saturate content decreases |G*| and T${}_{g}$ and increases the phase angle.
- A higher asphaltene content increases |G*| and decreases the phase angle.
- The aromatics and resin content does not correlate with |G*|, T${}_{g}$, or the phase angle, which may be due to them exhibiting opposite effects and being correlated in the considered data set.

Polymer modified bitumen:

- The amount of swelling is found to be determined by the saturate content of bitumen (primarily, saturates migrate into the polymer-rich phase, causing swelling).
- The polymer-rich phase content is stable or increases with temperature, resulting in improved phase stability at higher temperatures for higher saturate contents.
- At a low saturate content, T${}_{g}$ increases compared to the respective base bitumen due to the depletion of low molecular weight components and therefore the increase of high molecular weight components in the bitumen-rich matrix. At high saturate content, T${}_{g}$ decreases compared to the respective base bitumen due to phase inversion (the polymer-rich phase exhibits a lower glass transition temperature than the bitumen-rich phase).
- The |G*| of PmBs with a low saturate content increases compared to the respective base bitumen due to the saturate depletion of the bitumen-rich matrix phase.
- The |G*| of PmBs with a high saturate content decreases for lower temperatures and increases for higher temperatures compared to the respective base bitumen since the polymer-rich matrix phase exhibits a lower |G*| at lower temperatures and a higher |G*| at higher temperatures.
- The |G*| of the polymer-rich phase is decreased compared to pure polymer due to saturates’ incorporation.
- Phase angle δ decreases compared to the respective base bitumen for PmBs with a low saturate content due to the polymer-rich phase acting as an elastic filler in the bitumen-rich matrix phase.
- A high saturate content increases the PmB phase angle for lower temperatures and decreases for higher temperatures compared to the respective base bitumen. This is due to saturates incorporated by the polymer-rich phase reducing the phase angle of the polymer-rich phase for low temperatures, the polymer-rich phase being the matrix phase. At higher temperatures, the polymer-rich phase retains its elastic properties, thus reducing the phase angle compared to the crude bitumen.

## Figures and Tables

**Table 1:** Penetration grade and SARA fractions of the considered bitumens.

Bitumen Code	Penetration Grade	Saturates (%)	Aromatics (%)	Resins (%)	Asphaltenes (%)
A1	20/30	2.4	49.2	22.3	26.1
A2	30/45	3.9	35.2	35.2	25.7
B1	50/70	3.7	54.9	22.9	18.6
B2	70/100	4.8	42.3	31.8	21
C1	70/100	6.5	54.2	19.9	19.4
C2	160/220	7.5	42.7	30.3	19.5

**Table 2:** Temperature of equal |G*| for crude bitumen and polymer.

Crude Bitumen	Crude Bitumen T${}_{g}$ (°C)	PmB T${}_{g}$ (°C)
A1	−22.6	−2.5
A1	−18.2	−17.0
B1	−15.4	−13.3
B2	−11	−15.0
C1	−22.7	−29.8
C2	−26.7	−27.8

**Table 3:** Temperature where |G*| is equal for crude bitumen and polymer. For temperatures lower than the values given, the polymer |G*| is lower than the bitumen |G*| and higher for higher temperatures.

Crude Bitumen	Temperature (°C)
A1	20
A2	18
B1	15
B2	15
C1	10
C2	6

## Data Availability

Data are contained within the article or Supplementary Material. The data presented in this study are available in Supplementary File S1.

## Supplementary Materials

The following are available online at https://www.mdpi.com/1996-1944/14/5/1273/s1.
